# Associations of novel 24-h accelerometer-derived metrics with adiposity in children and adolescents

**DOI:** 10.1186/s12199-021-00987-5

**Published:** 2021-06-12

**Authors:** Jan Dygrýn, María Medrano, Pablo Molina-Garcia, Lukáš Rubín, Lukáš Jakubec, David Janda, Aleš Gába

**Affiliations:** 1 Faculty of Physical Culture, Palacký University Olomouc, třída Míru 117, 771 11 Olomouc, Czech Republic; 2 Faculty of Science, Humanities and Education, Technical University of Liberec, Liberec, Czech Republic

**Keywords:** Measurement, Accelerometer, Health, Body composition, Youth

## Abstract

**Background:**

Further research is required to explore the associations between 24-h movement behaviours and health outcomes in the paediatric population. Therefore, this study aimed to examine the associations between novel data-driven 24-h activity metrics and adiposity among children and adolescents.

**Methods:**

The sample included 382 children (8–13 years) and 338 adolescents (14–18 years). The average acceleration (AvAcc) of activity, intensity gradient (IG), and metrics representing the initial acceleration for the most active time periods of the 24-h cycle were calculated from raw acceleration data. Adiposity measures included body mass index *z*-score, fat mass percentage (FM%), and visceral adipose tissue (VAT). Data analysis was performed using multiple linear regression adjusted for wear time, sex, maternal education level, and maternal overweight and obesity.

**Results:**

Children demonstrated higher values in all 24-h activity metrics than did adolescents (*p* < 0.001 for all). For children, the initial acceleration for the most active 2, 5, 15, and 30 min of the 24-h cycle were negatively associated with FM% (*p* ≤ 0.043 for all) and VAT (*p* <0.001 for all), respectively. For adolescents, the IG was negatively associated with FM% (*p* = 0.002) and VAT (*p* = 0.007). Moreover, initial acceleration for the most active 2, 5, 15, 30, 60, and 120 min were associated with FM% (*p* ≤ 0.007 for all) and with VAT (*p* ≤ 0.023 for all).

**Conclusions:**

The intensity distribution of activity and initial acceleration for the most active 2, 5, 15, 30, 60, and 120 min within the 24-h cycle are beneficial for the prevention of excess adiposity in the paediatric population.

## Background

Childhood obesity is associated with several health outcomes [1, 2], social disadvantages [3], and long-term implications later in life [4]. According to the World Health Organization, the global prevalence of overweight and obesity has risen from 4% in 1975 to 18% in 2016 among children and adolescents [5]. Although there is evidence of a favourable trend in some countries in the recent years [5], the worldwide prevalence of childhood obesity is expected to increase, highlighting the necessity for effective multi-level and multi-sector prevention strategies [6].

In recent decades, evidence of non-modifiable and modifiable risk factors contributing to childhood obesity has rapidly increased. Research suggests that lack of sleep, excessive sedentary behaviour, and insufficient physical activity (PA) are independent risk factors for childhood obesity [7, 8]. Moreover, recent evidence has highlighted that these 24-h movement behaviours interact with each other and have cumulative effects on health [9, 10]. However, the simplification of 24-h activities to a few intensity categories leads to loss of information from continuous and complex human movement behaviours [11] and may affect their associations with health outcomes.

A new approach in data processing leads to the establishment of novel continuous data-driven analytical metrics (e.g., average acceleration (AvAcc), intensity gradient (IG), and metrics representing the initial acceleration for the most active X minutes (MX metrics) of the 24-h cycle) [12–14], which may provide unique insights into the role of 24-h movement behaviours in obesity prevention. The evaluation of movement behaviour using these metrics is not affected by decision of age- and sex-specific cut points for various intensities of PA and represent standardised, meaningful, and comparable acceleration metrics [13]. These new metrics are applicable only to multi-day high-resolution raw accelerometer data collected by new generations of research-grade accelerometers. Owing to recent technological progress (e.g., lower price, increasing battery capacity, and memory size), such types of accelerometers are gaining popularity even in large-sample studies under free-living conditions [11, 15].

Although previous studies have revealed that AvAcc, IG, and MX metrics are associated with several health indicators, including adiposity [16–19], no research has investigated these associations in a large sample of children and adolescents. Moreover, most previous studies used only body mass index (BMI) as an indicator of obesity, which has low sensitivity in identifying individuals with excess adiposity [20, 21]. Thus, the objective of this study was to analyse the cross-sectional associations between the novel 24-h accelerometer-derived metrics and directly measured adiposity in children and adolescents.

## Methods

### Participants

Children and adolescents aged 8–18 years from Czech Republic participated in this study. The procedure of data collection has been described previously in detail [10, 22]. In brief, data collection was conducted at school sites between 2018 and 2019. The main inclusion criteria were participant age and good health condition, as reported by their parents. Accelerometer data from 862 participants were available for data processing, regarding which 69 failed to meet the accelerometer wear-time inclusion criteria, and 73 did not undertake adiposity measurements. Thus, the final study sample comprised 720 participants.

### Accelerometer-derived 24-h activity metrics

ActiGraph accelerometers (GT9X Link or wGT3X-BT; ActiGraph LLC, Pensacola, FL, USA) were used to measure movement behaviours. Children and adolescents were instructed to wear a device on the non-dominant wrist for 24 h a day over 7 consecutive days, except for any activity that would involve its submergence in water for a prolonged period (such as bathing or swimming). The ActiLife software (ActiGraph LLC, Pensacola, FL, USA) was used to perform the following: (1) initialise devices to collect data on three axes at a frequency of 100 Hz, (2) download raw data in the GT3X format, and (3) convert data from the GT3X file to the .*csv* file format. These .*csv* files were analysed using the R package GGIR (v2.1-0, https://cran.r-project.org/web/packages/GGIR/). This procedure is described in detail elsewhere [23]. The default setting of R package GGIR and outputs only from ‘Part 2’ were used in this study.

The following 24-h movement behaviour metrics were obtained: AvAcc, IG, and the MX metrics, specifically the most active 2, 5, 15, 30, 60, and 120 min (M2, M5, M15, M30, M60, and M120, respectively) and the most active 8 h (M1/3_Day_) of the 24-h cycle [14, 24]. The AvAcc (expressed in milli-gravitational units (m*g*)) reflects directly measured dynamic acceleration over the 24-h cycle and presents a single metric for a total volume of activity. The IG reflects the distribution of acceleration intensity across the 24-h cycle and has been described in detail elsewhere [12, 14]; briefly, it illustrates the negative curvilinear relationship between the PA intensity and the time accumulated at that intensity during the 24-h cycle. All metrics were calculated separately for weekdays and weekend days and subsequently averaged (weighted) per day. Participants with at least 4 valid days (≥16 h/day, including at least 1 weekend day) [25], valid data available for all 15-min windows per 24-h cycle, and accelerometer post-calibration error (≤0.01 *g*) were included in the analysis [23].

### Adiposity indicators

The adiposity indicators used in this study were BMI *z*-score, fat mass percentage (FM%), and visceral adipose tissue (VAT). Body weight was measured using the InBody 720 device (InBody 720; Biospace Co, Ltd, ‘InBody’, Seoul, South Korea) to the nearest 0.1 kg and body height using Anthropometer P-375 (Trystom, Olomouc, Czech Republic) to the nearest 0.1 cm following standard measurement procedure. Based on sex- and age-specific BMI *z*-scores [26], children and adolescents were classified as follows: (1) underweight and normal weight (BMI *z*-score <1 standard deviation (SD)) and (2) overweight and obese (BMI *z*-score ≥1 SD). FM% and VAT were measured using the multi-frequency bioelectrical impedance method (InBody 720, 1–1 000 kHz) following the standard measurement protocol. The validity of multi-frequency bioimpedance analysis for assessing adiposity in the paediatric population has been confirmed [27].

### Covariates

In line with previous research [28] and based on stepwise preliminary analysis, we selected several covariates to control for potential confounding factors. The set of covariates included participant sex, accelerometer wear time (minutes per day), maternal education level, and maternal weight status. Maternal education level was recorded in 8 categories and dichotomized (0 = non-university level, 1 = university level) for the analysis. Maternal overweight/obesity was defined as a BMI ≥25 kg/m^2^, which was calculated using self-reported weight and height. Missing maternal education level (*n* = 25) and maternal height (*n* = 49) data were obtained by multiple imputations using the following predictor variables: maternal and paternal education levels (dummy variables), school location (dummy variables), BMI, BMI *z*-score, FM%, fat mass index, and fat-free mass index.

### Statistical analyses

Prior to all analyses, data were examined to detect extreme values and winsorized to reduce their influence. As there was an interaction between age and 24-h activity metrics for adiposity (*p* < 0.05), children and adolescents were analysed separately. Descriptive statistics were calculated for each variable and presented as mean (SD) for linear variables and frequencies for categorical variables. The chi-square test and Student’s *t*-test were used to calculate the differences between age categories. Means for MX metrics were plotted on a radar plot to visualise and interpret differences between children and adolescents [13]. Associations between the 24-h activity metrics and adiposity markers were assessed using linear regressions adjusted for all covariates. Multicollinearity was tested using pairwise correlation, tolerance, and variance inflation factor. There was no multicollinearity for models that included AvAcc and IG. A high risk of multicollinearity was identified for models with the MX metrics. Thus, the relationship between 24-h activity metrics and adiposity was analysed using (1) models that included both AvAcc and IG and (2) models in which each of MX metrics was included as a single independent variable. Such an approach is in the line with the GRANADA Consensus Statement which has been published recently [14]. Analyses were conducted using IBM SPSS Statistics version 25 (IBM, Armonk, NY, USA), and the level of significance was set at *p* < 0.05. The sample sizes of both age categories were sufficient to detect at least medium effect sizes and ensure a statistical power of ≥ 95% and an alpha error of 0.05 for regression models with five covariates [29].

## Results

**Fig. 1:**
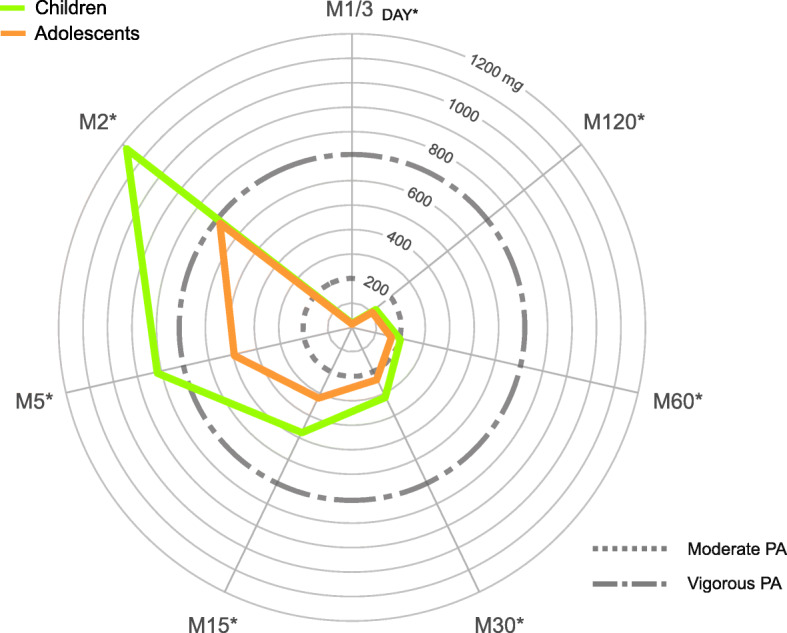
Radar plots illustrating differences in MX metrics between children and adolescents. MX metrics representing the acceleration (m*g*) above which the most active 2 min (M2), 5 min (M5), 15 min (M15), 30 min (M30), 60 min (M60), 120 min (M120), and 8 h (M1/3_DAY_) of the day are accumulated; PA, physical activity; *p < 0.05

Table 1 presents the characteristics of the study participants and shows how they vary across age categories. Children achieved significantly higher AvAcc (by 9.1 m*g*) and IG (by 0.23) units (*p* < 0.001 for all) than did adolescents. All MX metrics were significantly higher (*p* < 0.001 for all) in children than those in adolescents. Relative differences in the MX metrics between the age groups decreased with the duration of the most active period (Fig. 1). The mean M2 value demonstrated that acceleration in children was higher by 70.6% than that in adolescents, whilst the mean M1/3_Day_ value was higher by 16.6%. In all adiposity indicators, only difference (*p* < 0.001) in VAT between children and adolescents was found. Participants included in the analysis each wore an accelerometer for 23.6 h and for a median of 7 days. Approximately 39% of the mothers reported a high education level, and 37% were classified as overweight or having obesity.

**Table 1:** Characteristics of all the participants, children, and adolescents and the differences between them

	All (*n =* 720)	Children (*n* = 382)	Adolescents (*n* = 338)	*p*-value
Age (years)	13.9 (2.8)	11.7 (1.6)	16.4 (1.3)	**<0.001**
Girls (% of *n*)	56.2	56.2	55.9	0.866
**Anthropometric variables**				
Height (cm)	160.5 (14.2)	151.6 (12.1)	170.5 (8.7)	**<0.001**
Weight (kg)	53.1 (15.4)	43.8 (11.5)	63.7 (12.0)	**<0.001**
BMI *z*-score	0.25 (1.09)	0.27 (1.15)	0.23 (1.02)	0.691
Overweight and obesity prevalence (% of *n*)	23.6	25.4	21.6	0.231
FM%	20.1 (8.7)	19.5 (8.2)	20.7 (9.2)	0.065
Visceral adipose tissue (cm^2^)	49.2 (31.5)	43.5 (27.8)	55.6 (34.2)	**<0.001**
**24-h activity metrics**				
AvAcc (m*g*)	37.3 (10.9)	41.6 (11.2)	32.5 (8.2)	**<0.001**
IG	–2.19 (0.20)	–2.09 (0.16)	–2.31 (0.18)	**<0.001**
MX metrics (m*g*)				
M2	951.0 (448.2)	1180.3 (434.4)	691.8 (297.0)	**<0.001**
M5	665.8 (315.7)	817.6 (322.0)	494.3 (199.5)	**<0.001**
M15	398.7 (174.0)	472.2 (189.2)	315.6 (105.2)	**<0.001**
M30	275.0 (98.7)	312.8 (108.8)	232.3 (63.1)	**<0.001**
M60	158.3 (51.3)	202.7 (55.5)	165.7 (37.5)	**<0.001**
M120	116.1 (30.0)	124.9 (30.8)	106.0 (25.6)	**<0.001**
M1/3_Day_	20.3 (6.3)	21.8 (6.5)	18.7 (5.7)	**<0.001**
**Accelerometer wear**				
Wear time (min/day)	1413.7 (41.1)	1414.1 (39.0)	1413.3 (43.4)	0.784
Valid days	6.9 (0.2)	6.9 (0.2)	7.0 (0.1)	**<0.001**

*BMI*, body mass index; *FM%*, fat mass percentage; *AvAcc*, average acceleration; *IG*, intensity gradient; *M2*, *M5*, *M15*, *M30*, *M60*, *M120*, and *M1/3*_*Day*_, the acceleration above which the most active 2, 5, 15, 30, 60, 120 and min, and 8 h are accumulated, respectively

Values are presented as mean (SD) or percentage. The differences between children and adolescents were evaluated using the chi-square test and Student’s *t*-test for categorical and continuous variables, respectively. Boldfaced values: *p* < 0.05

The associations of 24-h activity metrics with BMI *z*-score, FM%, and VAT adjusted for sex, wear time, maternal education level, and weight status are presented for children in Table 2 and for adolescents in Table 3. In children, the M2, M5, M15, and M30 metrics were negatively associated with FM% (*p* ≤ 0.043 for all) and VAT (*p* <0.001 for all). For example, increasing M30 values by 100 m*g* was associated with a decrease in FM% by 0.9% points, and with a decrease in VAT by 5.1 cm^2^, respectively. In adolescents, IG and all MX metrics were negatively associated with FM% (*p* ≤ 0.007 for all) and with VAT (*p* ≤ 0.007 for all), except for M1/3_Day_. For example, increasing M30 metrics by 100 m*g* was associated with a decrease in FM% by 2.3% points, and with a decrease in VAT by 9.1 cm^2^, respectively. Similarly, an increase in IG by 0.05 units was associated with a 0.4% decrease in FM% and with a 1.6 cm^2^ decrease in VAT. No significant associations were observed between the 24-h activity metrics and BMI *z-*scores in both children and adolescents.

**Table 2:** Linear regression analysis of associations between 24-h activity metrics and adiposity indicators among children (*n* = 382)

	BMI *z*-score			FM%			VAT		
	*B*	(95% CI)	*p*-value	*B*	(95% CI)	*p*-value	*B*	(95% CI)	*p*-value
**24-h activity metrics**									
AvAcc (m*g*)	0.00	(−0.01, 0.02)	0.940	−0.06	(−0.16, 0.04)	0.214	−0.29	(−0.62, −0.04)	0.086
IG	−0.19	(−1.22, 0.85)	0.720	−2.06	(−9.07, 4.95)	0.564	−18.73	(−43.15, 5.69)	0.132
**MX metrics (m*g*)**									
M2	0.000	(0.00, 0.00)	0.591	−0.002	(0.00, 0.00)	**0.043**	−0.014	(−0.20, −0.01)	**<0.001**
M5	0.000	(0.00, 0.00)	0.276	−0.004	(−0.01, −0.00)	**0.006**	−0.021	(−0.03, −0.01)	**<0.001**
M15	0.000	(0.00, 0.00)	0.263	−0.006	(−0.01, −0.00)	**0.007**	−0.034	(−0.05, −0.02)	**<0.001**
M30	0.000	(0.00, 0.00)	0.577	−0.009	(−0.02, −0.00)	**0.025**	−0.051	(−0.08, −0.03)	**<0.001**
M60	0.000	(0.00, 0.00)	0.983	−0.013	(−0.03, 0.00)	0.074	−0.080	(−0.13, −0.03)	**0.002**
M120	0.001	(0.00, 0.00)	0.753	−0.023	(−0.05, 0.00)	0.086	−0.122	(−0.21, −0.03)	**0.008**
M1/3_Day_	0.003	(−0.01, 0.02)	0.721	−0.063	(−0.19, 0.06)	0.308	−0.419	(−0.85, 0.01)	0.055

*BMI*, body mass index; *FM%*, fat mass percentage; *VAT*, visceral adipose tissue; *B*, unstandardized beta; *CI*, confidence interval; *AvAcc*, average acceleration; *IG*, intensity gradient; *MX metrics* (*M2*, *M5*, *M15*, *M30*, *M60*, *M120*, and *M1/3*_*Day*_), the acceleration above which the most active 2, 5, 15, 30, 60, and 120 min, and 8 h are accumulated, respectively

Analyses were adjusted for sex, maternal education level, maternal overweight and obesity, and wear time. The model with average acceleration was in addition adjusted for intensity gradient and vice versa. Boldfaced values: *p* < 0.05

**Table 3:** Linear regression analysis of associations between 24-h activity metrics and adiposity indicators among adolescents (*n* = 338)

	BMI *z*-score			FM%			VAT		
	*B*	(95% CI)	*p*-value	*B*	(95% CI)	*p*-value	*B*	(95% CI)	*p*-value
**24-h activity metrics**									
AvAcc (m*g*)	0.00	(−0.01, 0.02)	0.822	−0.04	(−0.16, 0.07)	0.469	−0.17	(−0.68, 0.35)	0.522
IG	−0.25	(−0.99, 0.49)	0.512	−8.35	(−13.61, −3.09)	**0.002**	−32.5	(−55.98, −8.92)	**0.007**
MX metrics (m*g*)									
M2	0.000	(0.00, 0.00)	0.524	−0.006	(−0.01, −0.00)	**<0.001**	−0.025	(−0.04, −0.01)	**<0.001**
M5	0.000	(0.00, 0.00)	0.655	−0.008	(−0.01, −0.00)	**<0.001**	−0.033	(−0.05, −0.02)	**<0.001**
M15	0.000	(0.00, 0.00)	0.633	−0.014	(−0.02, −0.01)	**<0.001**	−0.059	(−0.09, −0.03)	**<0.001**
M30	0.000	(0.00, 0.00)	0.717	−0.023	(−0.03, −0.01)	**<0.001**	−0.091	(−0.15, −0.04)	**<0.001**
M60	0.000	(0.00, 0.00)	0.934	−0.034	(−0.05, −0.01)	**0.001**	−0.132	(−0.22, −0.04)	**0.005**
M120	0.001	(0.00, 0.01)	0.753	−0.041	(−0.07, −0.01)	**0.007**	−0.156	(−0.29, −0.02)	**0.023**
M1/3_Day_	0.005	(−0.01, 0.02)	0.604	−0.073	(−0.21, 0.06)	0.292	−0.282	(−0.89, −0.32)	0.358

*BMI*, body mass index; *FM%*, fat mass percentage; *VAT*, visceral adipose tissue; *B*, unstandardized beta; *CI*, confidence interval; *AvAcc*, average acceleration; *IG*, intensity gradient; *MX metrics* (*M2*, *M5*, *M15*, *M30*, *M60*, *M120*, and *M1/3Day*), the acceleration above which the most active 2, 5, 15, 30, 60, and 120 min, and 8 h are accumulated, respectively

Analyses were adjusted for sex, maternal education level, maternal overweight and obesity, and wear time. The model with average acceleration was in addition adjusted for intensity gradient and vice versa. Boldfaced values: p < 0.05

## Discussion

This is the first study to explore the associations between novel continuous data-driven analytical metrics and direct measures of adiposity in a large sample of children and adolescents. Our findings suggest that children have better 24-h activity profiles than adolescents. The M2, M5, M15, and M30 values were negatively (favourable) associated with FM% and VAT in both children and adolescents. Although IG was found to be negatively associated with FM% and VAT in adolescents, this was not observed in children.

In accordance with previous research [30, 31], our study found that children are more physically active than adolescents. Children achieved higher values in volume and intensity distribution of activity within a 24-h cycle. Similarly, children achieved higher acceleration in all MX metrics. The PA level of adolescents could be considered insufficient, as their mean value of the M60 metric did not exceed the 201 m*g* threshold, which is an alternative representation of the 60 min of the moderate-to-vigorous PA recommended for children and adolescents [32]. Similarly, our results also indicated that adolescents did not spend a substantial amount of time on vigorous PA because they did not achieve the estimated threshold of 707 m*g* [32], even in the most active 2 min of the day. It appears that children’s movement behaviours are characterised by a higher number of brief bouts of vigorous PA [33] such as spontaneous running or very fast movements during active play, than those of adolescents.

The independent negative associations between the IG and adiposity indicators were found among adolescents, but not children. This suggests that intensity distribution of activity is probably more important in the prevention of excess adiposity for adolescents than for children. It is known that the IG is designed to be sensitive to even very small amounts of high-intensity PA [34]. Sporadic high-intensity PA is considered to be higher in younger ages [31]. This may indicate that IG does not properly characterise the intensity distribution among highly active individuals. Our assumption needs to be confirmed by further research using various methods, such as multivariate pattern analysis [19], for assessing the PA intensity spectrum among groups with various intensity levels.

Our results suggest that the volume of activity represented by AvAcc did not predict adiposity in both age groups. Although this finding is in the line with the previous studies [17, 34], Rowlands et al. [34] suggest that AvAcc is an important contributor to adiposity only in 13–14 years girls. The inconsistency in the findings appears primarily due to variations in sex and age groups that have been investigated. We studied two age groups with both sexes, whilst Rowlands et al. [34] split the sample into groups with narrower age range and without boys in two of three age groups. Further studies allowing splitting the sample into several age categories and to analysing boys and girls separately is needed to understand the role of age and gender in the association between 24-h accelerometer-derived metrics with adiposity. Moreover, as AvAcc represents the average volume of activities accumulated per 24 h, it is naturally affected by acceleration occurring predominantly in activities such as sedentary behaviour and light PA. The patterns and context of these low-intensity activities and their associations with adiposity are currently the subject of intense research efforts [35–37]. The contextual role of AvAcc (e.g., AvAcc accumulated during wake-up or school-time periods) in association with adiposity should be further investigated in future studies to provide more evidence.

We are not aware of previous studies that have used MX metrics for investigating the associations between movement behaviour and adiposity among the paediatric population. Our study found a negative (favourable) association between MX metrics up to 30 min and FM% in both children and adolescents; these associations but for MX metrics up to 120 min were also observed for VAT. The MX metrics are easier to interpret in comparison with AvAcc or IG and have potential for public health messages [13]. For example, the values for M30 metrics were close to the thresholds for fast walking (~300 m*g*; 6.5 km/h) [32, 38] and brisk walking (~200 m*g*; 5 km/h) [32, 38] in children and adolescents, respectively. Thus, increasing the minimal acceleration of M30 by 100 m*g* is achievable for both children and adolescents, as this intensity still represents a moderate PA. From these results, it may be concluded that higher intensities, even performed in short periods of time, are more associated with adiposity than longer, less-intense periods in children and adolescents. This could also partially explain the non-association between IG and adiposity in children.

No significant associations were found between 24-h activity metrics and BMI *z-*scores in both children and adolescents. This is an unexpected finding, as other cross-sectional studies have shown a negative association of AvAcc and IG with BMI *z*-score [16, 19, 39, 40]. A plausible explanation for this finding may be that BMI has low sensitivity in identifying individuals with excess adiposity, especially in youth [20, 21].

The strengths of the present study are the application of multi-day high-resolution raw accelerometer data, the use of novel continuous data-driven analytical metrics, and the large sample size of children and adolescents. This study had several limitations. The cross-sectional design of the study precluded our ability to infer a causal relationship between 24-h activity metrics and adiposity in children and adolescents. Longitudinal and interventional studies are required to confirm the associations observed in this study. Our study was also limited by the lack of data on screen time and socioeconomic status, which might have affected the associations.

## Conclusion

In conclusion, the higher initial acceleration in MX metrics up to 30 and up to 120 min is beneficial for the prevention of excess adiposity among children and adolescents, respectively. The intensity distribution of activity for adolescents rather than the volume of activity may be more important for adiposity. Our findings contribute to the growing evidence supporting the use of novel data-driven accelerometer analysis and translation metrics in movement-behaviour research, especially in studies using wrist-worn accelerometers. Future studies employing longitudinal data are required to confirm the direction of causality.

## Data Availability

The dataset analysed during the current study is available in the Figshare repository, 10.6084/m9.figshare.14153312.

## Abbreviations

AvAcc: Average acceleration
IG: Intensity gradient
PA: Physical activity
MX: Most active X minutes
BMI: Body mass index
FM%: Fat mass percentage
VAT: Visceral adipose tissue
